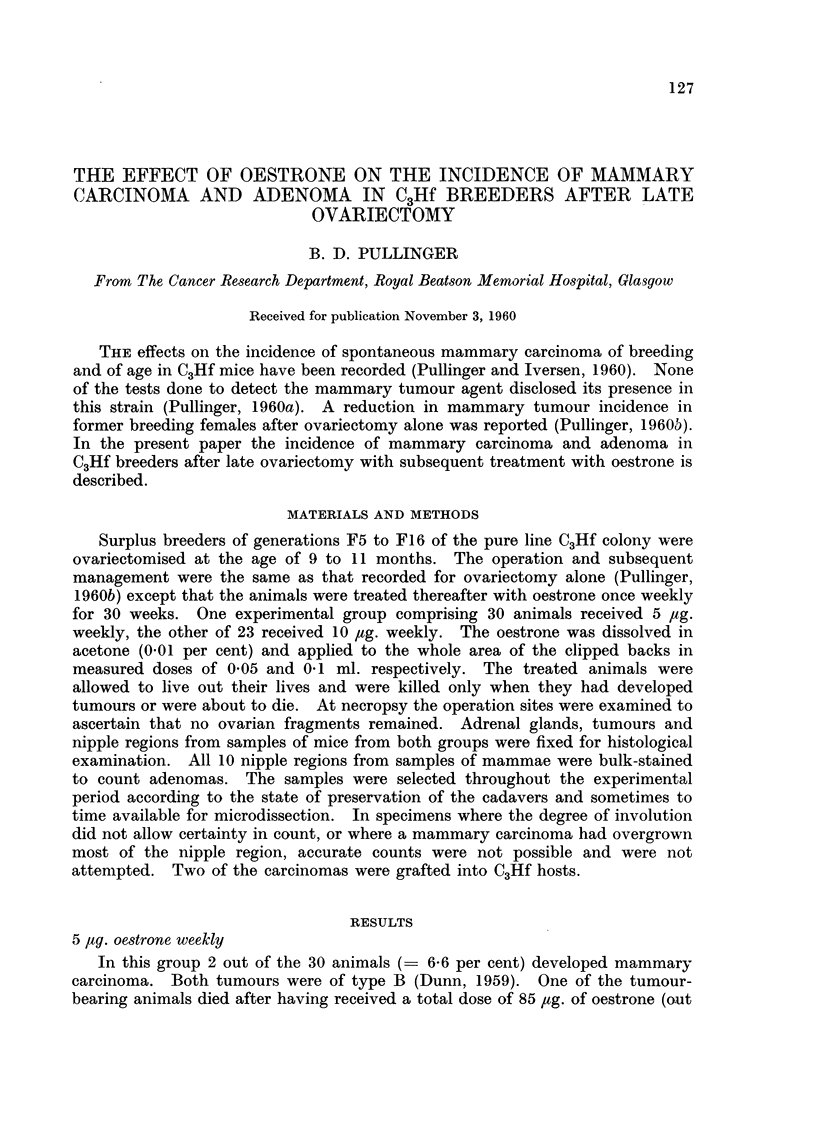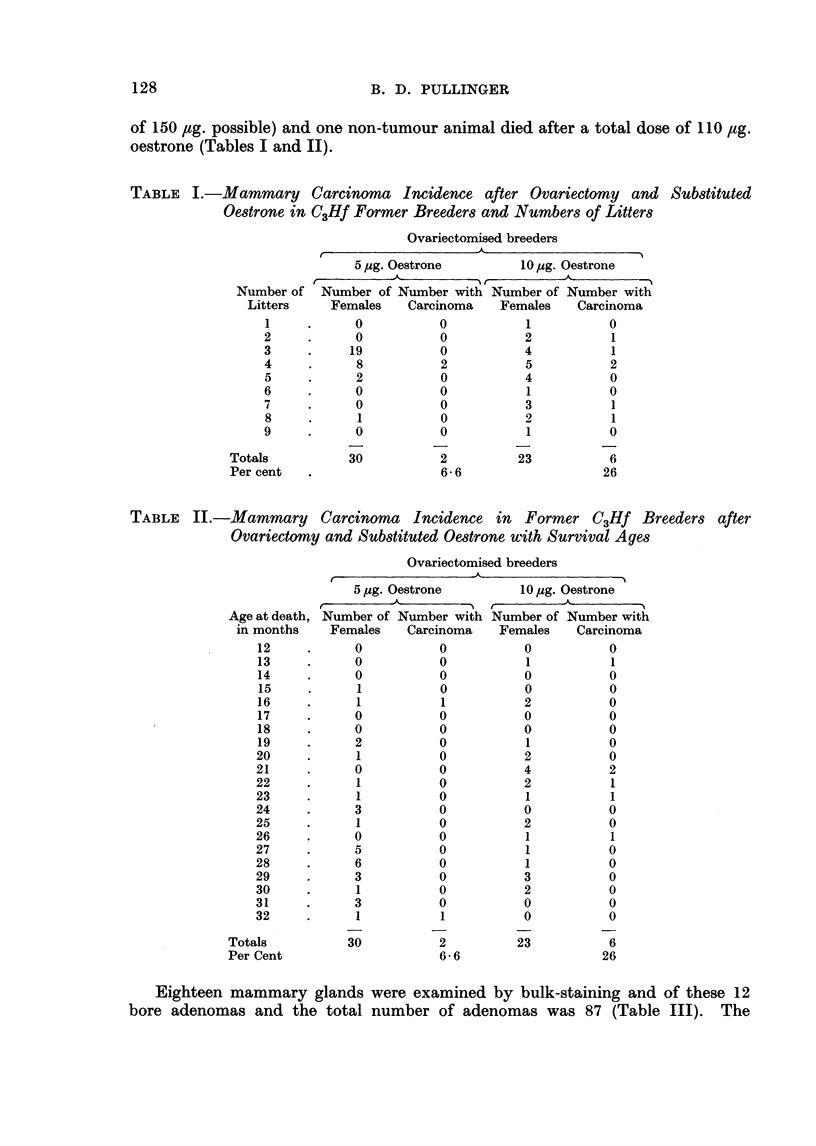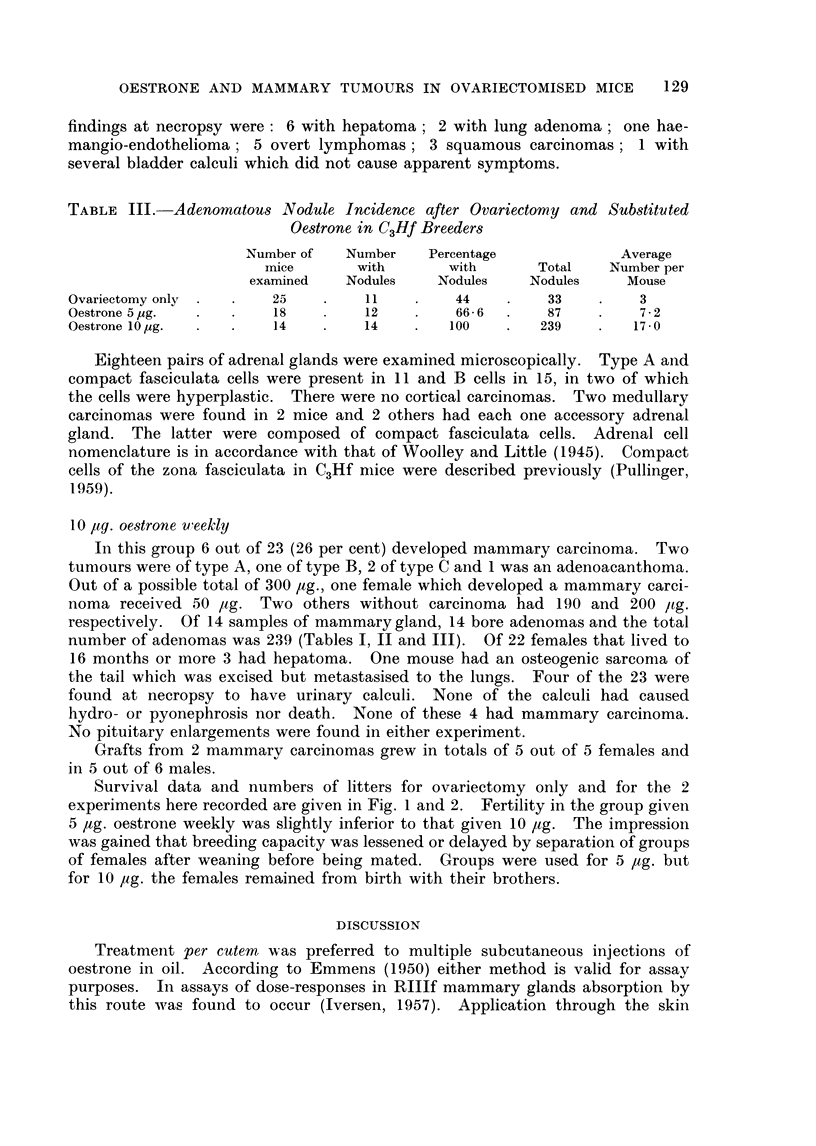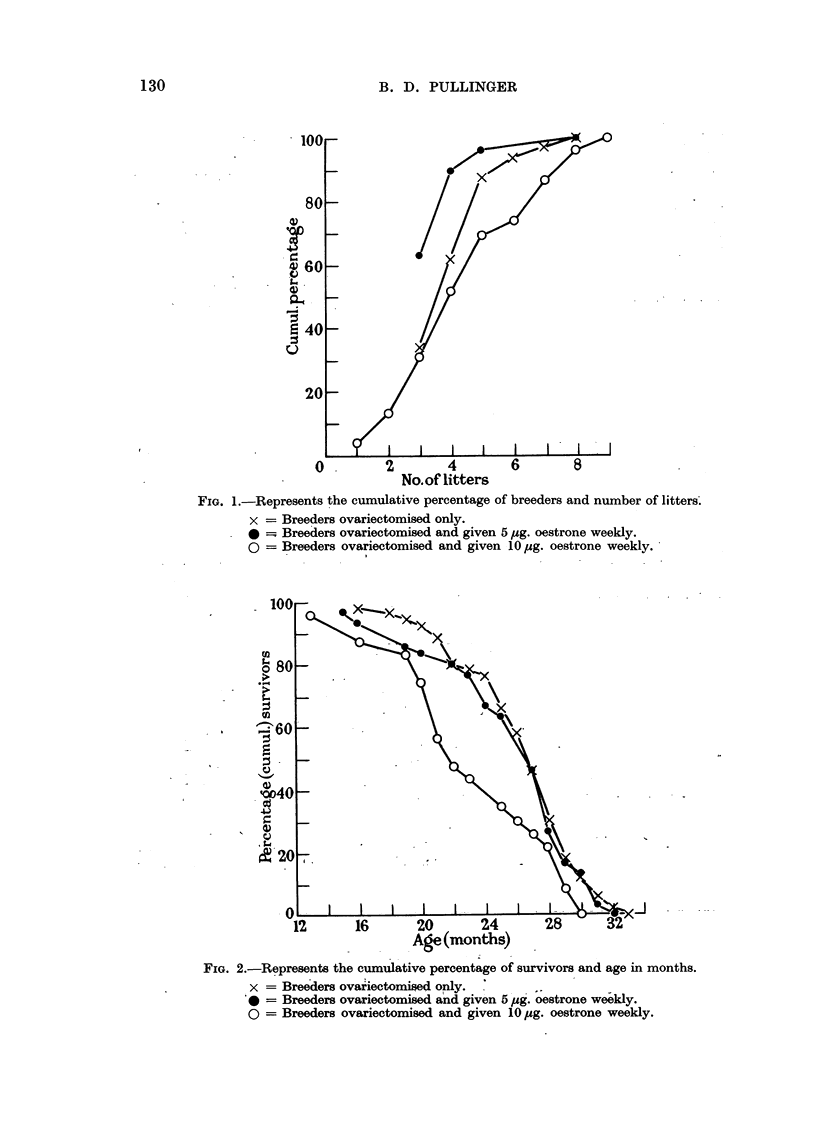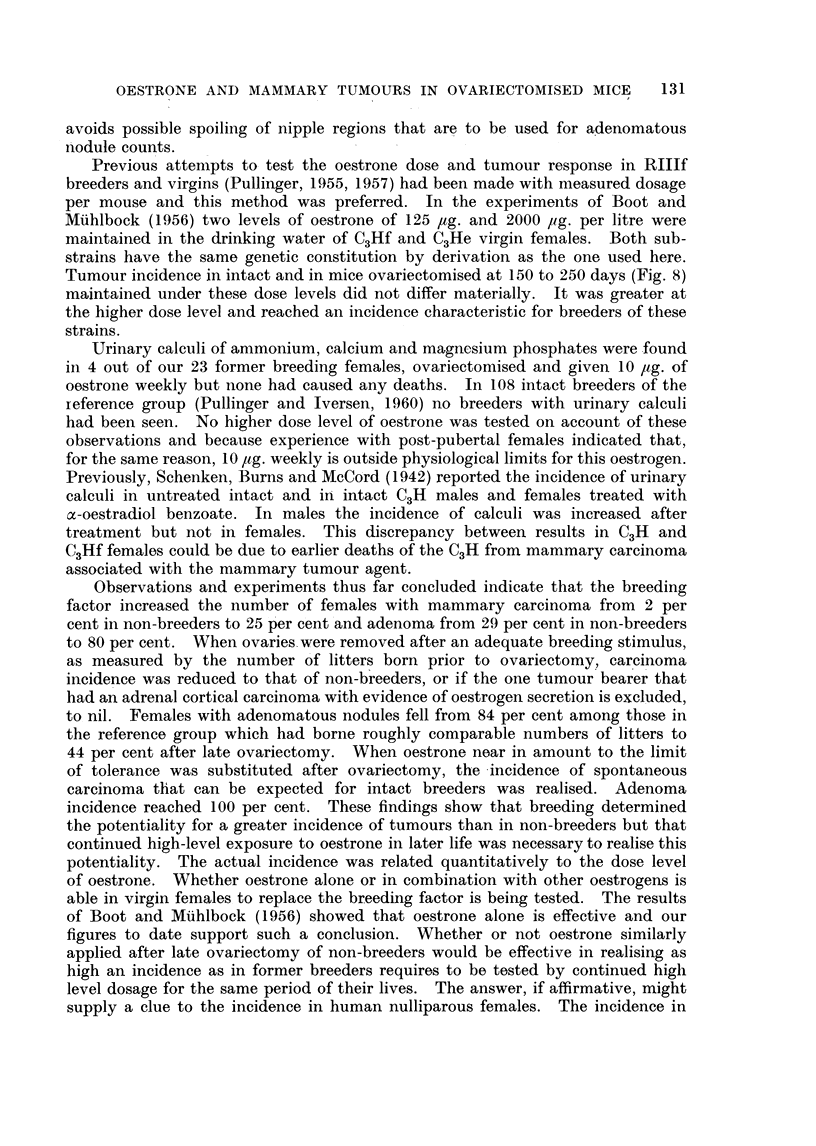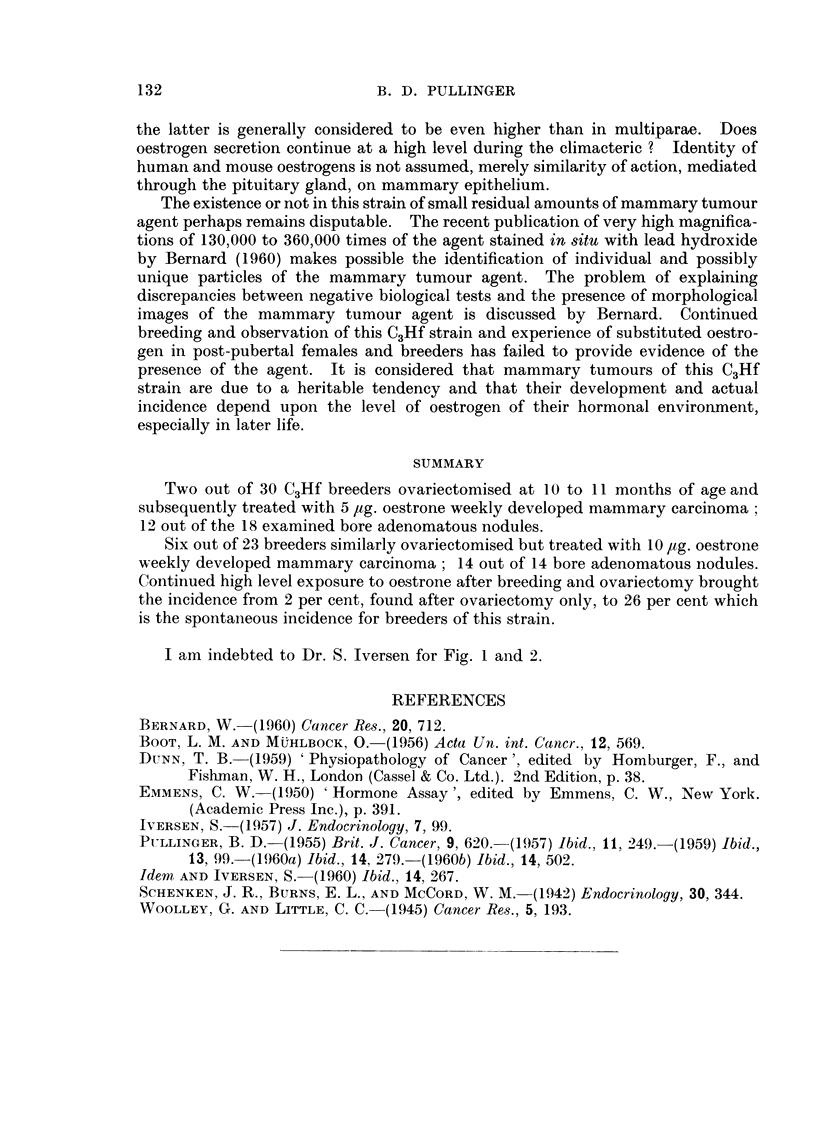# The Effect of Oestrone on the Incidence of Mammary Carcinoma and Adenoma in C_3_Hf Breeders After Late Ovariectomy

**DOI:** 10.1038/bjc.1961.15

**Published:** 1961-03

**Authors:** B. D. Pullinger


### THE EFFECT OF OESTRONE ON THE INCIDENCE OF MAMMARY CARCINOMA AND ADENOMA IN $C_3Hf$ BREEDERS AFTER LATE OVARIECTOMY

B. D. PULLINGER

*From The Cancer Research Department, Royal Beatson Memorial Hospital, Glasgow*

Received for publication November 3, 1960

THE effects on the incidence of spontaneous mammary carcinoma of breeding and of age in $C_3Hf$ mice have been recorded (Pullinger and Iversen, 1960). None of the tests done to detect the mammary tumour agent disclosed its presence in this strain (Pullinger, 1960*a*). A reduction in mammary tumour incidence in former breeding females after ovariectomy alone was reported (Pullinger, 1960*b*). In the present paper the incidence of mammary carcinoma and adenoma in $C_3Hf$ breeders after late ovariectomy with subsequent treatment with oestrone is described.

### MATERIALS AND METHODS

Surplus breeders of generations F5 to F16 of the pure line $C_3Hf$ colony were ovariectomised at the age of 9 to 11 months. The operation and subsequent management were the same as that recorded for ovariectomy alone (Pullinger, 1960*b*) except that the animals were treated thereafter with oestrone once weekly for 30 weeks. One experimental group comprising 30 animals received 5 $\mu$g. weekly, the other of 23 received 10 $\mu$g. weekly. The oestrone was dissolved in acetone (0·01 per cent) and applied to the whole area of the clipped backs in measured doses of 0·05 and 0·1 ml. respectively. The treated animals were allowed to live out their lives and were killed only when they had developed tumours or were about to die. At necropsy the operation sites were examined to ascertain that no ovarian fragments remained. Adrenal glands, tumours and nipple regions from samples of mice from both groups were fixed for histological examination. All 10 nipple regions from samples of mammae were bulk-stained to count adenomas. The samples were selected throughout the experimental period according to the state of preservation of the cadavers and sometimes to time available for microdissection. In specimens where the degree of involution did not allow certainty in count, or where a mammary carcinoma had overgrown most of the nipple region, accurate counts were not possible and were not attempted. Two of the carcinomas were grafted into $C_3Hf$ hosts.

### RESULTS

### 5 $\mu$g. oestrone weekly

In this group 2 out of the 30 animals (= 6·6 per cent) developed mammary carcinoma. Both tumours were of type B (Dunn, 1959). One of the tumour-bearing animals died after having received a total dose of 85 $\mu$g. of oestrone (out of 150 $\mu$g. possible) and one non-tumour animal died after a total dose of 110 $\mu$g. oestrone (Tables I and II).

TABLE I.—*Mammary Carcinoma Incidence after Ovariectomy and Substituted Oestrone in $C_3Hf$ Former Breeders and Numbers of Litters*

| | Ovariectomised breeders | | | |
|---|---|---|---|---|
| | 5 $\mu$g. Oestrone | | 10 $\mu$g. Oestrone | |
| Number of Litters | Number of Females | Number with Carcinoma | Number of Females | Number with Carcinoma |
| 1 | 0 | 0 | 1 | 0 |
| 2 | 0 | 0 | 2 | 1 |
| 3 | 19 | 0 | 4 | 1 |
| 4 | 8 | 2 | 5 | 2 |
| 5 | 2 | 0 | 4 | 0 |
| 6 | 0 | 0 | 1 | 0 |
| 7 | 0 | 0 | 3 | 1 |
| 8 | 1 | 0 | 2 | 1 |
| 9 | 0 | 0 | 1 | 0 |
| Totals | 30 | 2 | 23 | 6 |
| Per cent | | 6·6 | | 26 |

TABLE II.—*Mammary Carcinoma Incidence in Former $C_3Hf$ Breeders after Ovariectomy and Substituted Oestrone with Survival Ages*

| | Ovariectomised breeders | | | |
|---|---|---|---|---|
| | 5 $\mu$g. Oestrone | | 10 $\mu$g. Oestrone | |
| Age at death, in months | Number of Females | Number with Carcinoma | Number of Females | Number with Carcinoma |
| 12 | 0 | 0 | 0 | 0 |
| 13 | 0 | 0 | 1 | 1 |
| 14 | 0 | 0 | 0 | 0 |
| 15 | 1 | 0 | 0 | 0 |
| 16 | 1 | 1 | 2 | 0 |
| 17 | 0 | 0 | 0 | 0 |
| 18 | 0 | 0 | 0 | 0 |
| 19 | 2 | 0 | 1 | 0 |
| 20 | 1 | 0 | 2 | 0 |
| 21 | 0 | 0 | 4 | 2 |
| 22 | 1 | 0 | 2 | 1 |
| 23 | 1 | 0 | 1 | 1 |
| 24 | 3 | 0 | 0 | 0 |
| 25 | 1 | 0 | 2 | 0 |
| 26 | 0 | 0 | 1 | 1 |
| 27 | 5 | 0 | 1 | 0 |
| 28 | 6 | 0 | 1 | 0 |
| 29 | 3 | 0 | 3 | 0 |
| 30 | 1 | 0 | 2 | 0 |
| 31 | 3 | 0 | 0 | 0 |
| 32 | 1 | 1 | 0 | 0 |
| Totals | 30 | 2 | 23 | 6 |
| Per Cent | | 6·6 | | 26 |

Eighteen mammary glands were examined by bulk-staining and of these 12 bore adenomas and the total number of adenomas was 87 (Table III). The findings at necropsy were : 6 with hepatoma ; 2 with lung adenoma ; one haemangio-endothelioma ; 5 overt lymphomas ; 3 squamous carcinomas ; 1 with several bladder calculi which did not cause apparent symptoms.

TABLE III.—*Adenomatous Nodule Incidence after Ovariectomy and Substituted Oestrone in $C_3Hf$ Breeders*

| | Number of mice examined | Number with Nodules | Percentage with Nodules | Total Nodules | Average Number per Mouse |
|---|---|---|---|---|---|
| Ovariectomy only | 25 | 11 | 44 | 33 | 3 |
| Oestrone 5 $\mu$g. | 18 | 12 | 66·6 | 87 | 7·2 |
| Oestrone 10 $\mu$g. | 14 | 14 | 100 | 239 | 17·0 |

Eighteen pairs of adrenal glands were examined microscopically. Type A and compact fasciculata cells were present in 11 and B cells in 15, in two of which the cells were hyperplastic. There were no cortical carcinomas. Two medullary carcinomas were found in 2 mice and 2 others had each one accessory adrenal gland. The latter were composed of compact fasciculata cells. Adrenal cell nomenclature is in accordance with that of Woolley and Little (1945). Compact cells of the zona fasciculata in $C_3$Hf mice were described previously (Pullinger, 1959).

*10 $\mu$g. oestrone weekly*

In this group 6 out of 23 (26 per cent) developed mammary carcinoma. Two tumours were of type A, one of type B, 2 of type C and 1 was an adenoacanthoma. Out of a possible total of 300 $\mu$g., one female which developed a mammary carcinoma received 50 $\mu$g. Two others without carcinoma had 190 and 200 $\mu$g. respectively. Of 14 samples of mammary gland, 14 bore adenomas and the total number of adenomas was 239 (Tables I, II and III). Of 22 females that lived to 16 months or more 3 had hepatoma. One mouse had an osteogenic sarcoma of the tail which was excised but metastasised to the lungs. Four of the 23 were found at necropsy to have urinary calculi. None of the calculi had caused hydro- or pyonephrosis nor death. None of these 4 had mammary carcinoma. No pituitary enlargements were found in either experiment.

Grafts from 2 mammary carcinomas grew in totals of 5 out of 5 females and in 5 out of 6 males.

Survival data and numbers of litters for ovariectomy only and for the 2 experiments here recorded are given in Fig. 1 and 2. Fertility in the group given 5 $\mu$g. oestrone weekly was slightly inferior to that given 10 $\mu$g. The impression was gained that breeding capacity was lessened or delayed by separation of groups of females after weaning before being mated. Groups were used for 5 $\mu$g. but for 10 $\mu$g. the females remained from birth with their brothers.

### DISCUSSION

Treatment *per cutem* was preferred to multiple subcutaneous injections of oestrone in oil. According to Emmens (1950) either method is valid for assay purposes. In assays of dose-responses in RIIIf mammary glands absorption by this route was found to occur (Iversen, 1957). Application through the skin

FIG. 1.—Represents the cumulative percentage of breeders and number of litters.
× = Breeders ovariectomised only.
● = Breeders ovariectomised and given 5 μg. oestrone weekly.
○ = Breeders ovariectomised and given 10 μg. oestrone weekly.

FIG. 2.—Represents the cumulative percentage of survivors and age in months.
× = Breeders ovariectomised only.
● = Breeders ovariectomised and given 5 μg. oestrone weekly.
○ = Breeders ovariectomised and given 10 μg. oestrone weekly.

avoids possible spoiling of nipple regions that are to be used for adenomatous nodule counts.

Previous attempts to test the oestrone dose and tumour response in RIIIf breeders and virgins (Pullinger, 1955, 1957) had been made with measured dosage per mouse and this method was preferred. In the experiments of Boot and Mühlbock (1956) two levels of oestrone of 125 $\mu$g. and 2000 $\mu$g. per litre were maintained in the drinking water of $C_3$Hf and $C_3$He virgin females. Both substrains have the same genetic constitution by derivation as the one used here. Tumour incidence in intact and in mice ovariectomised at 150 to 250 days (Fig. 8) maintained under these dose levels did not differ materially. It was greater at the higher dose level and reached an incidence characteristic for breeders of these strains.

Urinary calculi of ammonium, calcium and magnesium phosphates were found in 4 out of our 23 former breeding females, ovariectomised and given 10 $\mu$g. of oestrone weekly but none had caused any deaths. In 108 intact breeders of the reference group (Pullinger and Iversen, 1960) no breeders with urinary calculi had been seen. No higher dose level of oestrone was tested on account of these observations and because experience with post-pubertal females indicated that, for the same reason, 10 $\mu$g. weekly is outside physiological limits for this oestrogen. Previously, Schenken, Burns and McCord (1942) reported the incidence of urinary calculi in untreated intact and in intact $C_3$H males and females treated with $\alpha$-oestradiol benzoate. In males the incidence of calculi was increased after treatment but not in females. This discrepancy between results in $C_3$H and $C_3$Hf females could be due to earlier deaths of the $C_3$H from mammary carcinoma associated with the mammary tumour agent.

Observations and experiments thus far concluded indicate that the breeding factor increased the number of females with mammary carcinoma from 2 per cent in non-breeders to 25 per cent and adenoma from 29 per cent in non-breeders to 80 per cent. When ovaries were removed after an adequate breeding stimulus, as measured by the number of litters born prior to ovariectomy, carcinoma incidence was reduced to that of non-breeders, or if the one tumour bearer that had an adrenal cortical carcinoma with evidence of oestrogen secretion is excluded, to nil. Females with adenomatous nodules fell from 84 per cent among those in the reference group which had borne roughly comparable numbers of litters to 44 per cent after late ovariectomy. When oestrone near in amount to the limit of tolerance was substituted after ovariectomy, the incidence of spontaneous carcinoma that can be expected for intact breeders was realised. Adenoma incidence reached 100 per cent. These findings show that breeding determined the potentiality for a greater incidence of tumours than in non-breeders but that continued high-level exposure to oestrone in later life was necessary to realise this potentiality. The actual incidence was related quantitatively to the dose level of oestrone. Whether oestrone alone or in combination with other oestrogens is able in virgin females to replace the breeding factor is being tested. The results of Boot and Mühlbock (1956) showed that oestrone alone is effective and our figures to date support such a conclusion. Whether or not oestrone similarly applied after late ovariectomy of non-breeders would be effective in realising as high an incidence as in former breeders requires to be tested by continued high level dosage for the same period of their lives. The answer, if affirmative, might supply a clue to the incidence in human nulliparous females. The incidence in the latter is generally considered to be even higher than in multiparae. Does oestrogen secretion continue at a high level during the climacteric ? Identity of human and mouse oestrogens is not assumed, merely similarity of action, mediated through the pituitary gland, on mammary epithelium.

The existence or not in this strain of small residual amounts of mammary tumour agent perhaps remains disputable. The recent publication of very high magnifications of 130,000 to 360,000 times of the agent stained *in situ* with lead hydroxide by Bernard (1960) makes possible the identification of individual and possibly unique particles of the mammary tumour agent. The problem of explaining discrepancies between negative biological tests and the presence of morphological images of the mammary tumour agent is discussed by Bernard. Continued breeding and observation of this $C_3Hf$ strain and experience of substituted oestrogen in post-pubertal females and breeders has failed to provide evidence of the presence of the agent. It is considered that mammary tumours of this $C_3Hf$ strain are due to a heritable tendency and that their development and actual incidence depend upon the level of oestrogen of their hormonal environment, especially in later life.

### SUMMARY

Two out of 30 $C_3Hf$ breeders ovariectomised at 10 to 11 months of age and subsequently treated with 5 $\mu$g. oestrone weekly developed mammary carcinoma ; 12 out of the 18 examined bore adenomatous nodules.

Six out of 23 breeders similarly ovariectomised but treated with 10 $\mu$g. oestrone weekly developed mammary carcinoma ; 14 out of 14 bore adenomatous nodules. Continued high level exposure to oestrone after breeding and ovariectomy brought the incidence from 2 per cent, found after ovariectomy only, to 26 per cent which is the spontaneous incidence for breeders of this strain.

I am indebted to Dr. S. Iversen for Fig. 1 and 2.

### REFERENCES

BERNARD, W.—(1960) *Cancer Res.*, **20**, 712.
BOOT, L. M. AND MÜHLBOCK, O.—(1956) *Acta Un. int. Cancr.*, **12**, 569.
DUNN, T. B.—(1959) ‘ Physiopathology of Cancer ’, edited by Homburger, F., and Fishman, W. H., London (Cassel & Co. Ltd.). 2nd Edition, p. 38.
EMMENS, C. W.—(1950) ‘ Hormone Assay ’, edited by Emmens, C. W., New York. (Academic Press Inc.), p. 391.
IVERSEN, S.—(1957) *J. Endocrinology*, **7**, 99.
PULLINGER, B. D.—(1955) *Brit. J. Cancer*, **9**, 620.—(1957) *Ibid.*, **11**, 249.—(1959) *Ibid.*, **13**, 99.—(1960*a*) *Ibid.*, **14**, 279.—(1960*b*) *Ibid.*, **14**, 502.
*Idem* AND IVERSEN, S.—(1960) *Ibid.*, **14**, 267.
SCHENKEN, J. R., BURNS, E. L., AND MCCORD, W. M.—(1942) *Endocrinology*, **30**, 344.
WOOLLEY, G. AND LITTLE, C. C.—(1945) *Cancer Res.*, **5**, 193.